# Axonal Projections From the Middle Temporal Area in the Common Marmoset

**DOI:** 10.3389/fnana.2018.00089

**Published:** 2018-10-30

**Authors:** Hiroshi Abe, Toshiki Tani, Hiromi Mashiko, Naohito Kitamura, Taku Hayami, Satoshi Watanabe, Kazuhisa Sakai, Wataru Suzuki, Hiroaki Mizukami, Akiya Watakabe, Tetsuo Yamamori, Noritaka Ichinohe

**Affiliations:** ^1^Ichinohe Group, Laboratory for Molecular Analysis of Higher Brain Function, Center for Brain Science, RIKEN, Saitama, Japan; ^2^Department of Ultrastructural Research, National Institute of Neuroscience, National Center of Neurology and Psychiatry, Tokyo, Japan; ^3^Division of Genetic Therapeutics, Center for Molecular Medicine, Jichi Medical University, Tochigi, Japan; ^4^Laboratory for Molecular Analysis of Higher Brain Function, Center for Brain Science, RIKEN, Saitama, Japan

**Keywords:** marmoset, middle temporal area, MT, optical intrinsic signal imaging, projection, temporal cortex

## Abstract

Neural activity in the middle temporal (MT) area is modulated by the direction and speed of motion of visual stimuli. The area is buried in a sulcus in the macaque, but exposed to the cortical surface in the marmoset, making the marmoset an ideal animal model for studying MT function. To better understand the details of the roles of this area in cognition, underlying anatomical connections need to be clarified. Because most anatomical tracing studies in marmosets have used retrograde tracers, the axonal projections remain uncharacterized. In order to examine axonal projections from MT, we utilized adeno-associated viral (AAV) tracers, which work as anterograde tracers by expressing either green or red fluorescent protein in infected neurons. AAV tracers were injected into three sites in MT based on retinotopy maps obtained via *in vivo* optical intrinsic signal imaging. Brains were sectioned and divided into three series, one for fluorescent image scanning and two for myelin and Nissl substance staining to identify specific brain areas. Overall projection patterns were similar across the injections. MT projected to occipital visual areas V1, V2, V3 (VLP) and V4 (VLA) and surrounding areas in the temporal cortex including MTC (V4T), MST, FST, FSTv (PGa/IPa) and TE3. There were also projections to the dorsal visual pathway, V3A (DA), V6 (DM) and V6A, the intraparietal areas AIP, LIP, MIP, frontal A4ab and the prefrontal cortex, A8aV and A8C. There was a visuotopic relationship with occipital visual areas. In a marmoset in which two tracer injections were made, the projection targets did not overlap in A8aV and AIP, suggesting topographic projections from different parts of MT. Most of these areas are known to send projections back to MT, suggesting that they are reciprocally connected with it.

## Introduction

The middle temporal (MT) area or V5 was first defined by Allman and Kaas (1971) in the owl monkey, and has since been an extensively studied visual cortical area, especially in macaques (Born and Bradley, 2005; Lui and Rosa, 2015). Its neural activity represents the direction and speed of motion of visual stimuli (Dubner and Zeki, 1971; Maunsell and van Essen, 1983a; Albright, 1984; Felleman and Kaas, 1984). MT represents the entire contralateral visual field and incorporates a magnified representation of the central visual field (Desimone and Ungerleider, 1986) which is considered important for the perception of motion of observed stimuli. Other motion-sensitive areas such as MST and V6 (DM) also represent entire contralateral visual fields but with larger receptive fields (Desimone and Ungerleider, 1986; Galletti et al., 1999) and represent visual cues related to self-motion (Cardin and Smith, 2010; Pitzalis et al., 2013). Furthermore, combined with a psychophysics paradigm, analyses of MT function suggest a causal link to perception (Newsome and Paré, 1988; Salzman et al., 1990; Britten et al., 1996), giving rise to insights about neural coding and neural mechanisms of decision-making (Zohary et al., 1994; Shadlen et al., 1996). Thus, studies on MT functions have played an important role in cognitive neuroscience.

MT is present in all primates (Lui and Rosa, 2015). In the macaque it is located in a sulcus, but it is exposed to the cortical surface in marmosets. Indeed, the marmoset is the only simian primate in which MT is located entirely at the surface. Even in other similar species such as the squirrel monkey and owl monkey, only a part of MT is located at the surface. This feature makes the marmoset an ideal animal model for studying MT functions via several types of new techniques, including two-photon imaging and array electrode implantation (Chen et al., 2015; Townsend et al., 2015, 2017; Zavitz et al., 2016, 2017). To better understand the details of the roles of this area in cognition, underlying anatomical connections need to be clarified. Previous studies (Krubitzer and Kaas, 1990; Palmer and Rosa, 2006), pre-date the current comprehensive knowledge of boundaries of cortical areas in the marmoset (Burman et al., 2006, 2008; Rosa et al., 2009; Paxinos et al., 2012), so it is difficult to compare results in terms of currently known areas.

**Table 1:** Subject information.

Subject	Sex	Weight	Age at injection	Anesthetic during surgery
Marmoset 1	F	353 g	3 years 4 months	Sevoflurane 1%–2%
Marmoset 2	F	348 g	2 years 4 months	Isoflurane 1%–2%

## Materials and Methods

### Animals and Surgery

The experiments were performed in two marmosets (*Callithrix jacchus*; Table 1). All experimental procedures were approved by the Experimental Animal Committee of RIKEN, or by the Experimental Animal Committee of the National Center of Neurology and Psychiatry. The marmosets were handled in accordance with the “Guiding Principles of the Care and Use of Animals in the Field of Physiological Science” formulated by the Japanese Physiological Society.

**Figure 1:**
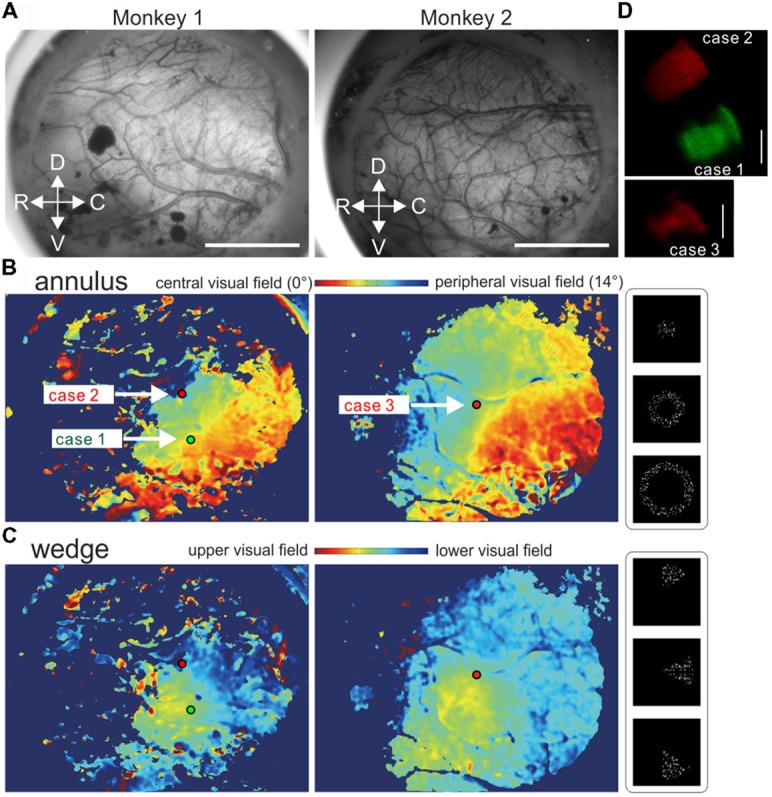
Virus injections based on retinotopy maps obtained using *in vivo* optical intrinsic signal imaging. **(A)** Cortical surface images through the intact dura in regions around the middle temporal (MT) area in the left hemispheres of marmosets 1 and 2. **(B,C)** Retinotopy maps and tracer injection sites. **(B,C)** Color-coded retinotopy maps of visual field locations estimated with the annulus **(B)** and wedge **(C)** apertures. The green and red dots indicate virus injection sites. **(D)** Zoomed-in images of parts of brain sections around the injection sites showing virus tracer spread. The bars indicate 5 mm and 1 mm in **(A,D)**, respectively.

We followed previously described experimental procedures (Suzuki et al., 2015a,b; Abe et al., 2017; Miyakawa et al., 2017). Food and water were withdrawn in the evening before the day of the experiment. Following atropine sulfate (0.15 μg/kg intramuscular) and ketamine hydrochloride (Ketalar, 25 mg/kg intramuscular) injections, aseptic surgery was conducted under anesthesia. The marmoset was intubated and artificially ventilated by a respirator. Anesthesia was maintained via 1%–2% isoflurane or sevoflurane with a mixture of 50% N_2_O and 50% O_2_. Electrocardiography, expired CO_2_, SpO_2_ and rectal temperature were monitored continuously throughout the experiment. The marmoset was placed in a stereotactic apparatus. A head holder was implanted on the skull. The head was fixed with the head holder and the stereotactic apparatus was removed, resulting in a space in front of the eyes for visual stimulation. A stainless-steel chamber (inner diameter 18 mm; Figure 1A) was implanted on the skull after craniotomy and filled with agar to reduce the effects of pulsation during imaging.

### *In vivo* Optical Intrinsic Imaging and Tracer Injection

To obtain a retinotopy map, optical intrinsic signal imaging was performed through the dura just before the tracer injection. The cortical surface was illuminated by a halogen lamp or an LED light (535-nm wavelength) and captured by a CCD camera (GRAS-03K2M-C, FLIR Integrated Imaging Solutions Inc., Richmond, BC, Canada) with a lens (Ai AF Micro-Nikkor 60-mm f/2.8D, Nikon, Tokyo, Japan) in a 640 × 480 pixel format at 30 Hz. The focal depth was set 600–800 μm below the cortical surface. Before imaging, gaseous anesthesia was switched to a combination of remifentanil (Ultiva, 0.1 μg/kg/min, intravenous) and rocuronium bromide (Eslax, 13 μg/kg/min, intravenous; Suzuki et al., 2015a,b; Abe et al., 2017; Miyakawa et al., 2017). The pupil was fully dilated with 0.5% topical tropicamide. A contact lens was used to focus at a distance of 57 cm for the eye contralateral to the imaged hemisphere (Suzuki et al., 2015a,b) with the aid of an ophthalmoscope (iExaminer, Welch Allyn, Skaneateles Falls, NY, USA), which was set on a custom holder with a rotating stage on a tripod. The fovea direction was back-projected on a computer monitor by rotating the stage 180° to align stimulus locations.

Moving dots were presented through an annulus (Figure 1B) or a wedge aperture (Figure 1C) in three or four different sizes for marmoset 1 or 2, respectively. The largest annulus and the largest wedge had diameters of 28°. Five-hundred white dots (100% Michelson contrast, 0.54° in diameter) moved incoherently in fixed random directions at a speed of 8.5°/s within a circular region with a 16-degree radius. Each dot was replaced with a new one with a probability of 10% per frame (1/60 s. For each trial, after a 2-s baseline period one of the stimuli was presented for 2 s followed by a 15-s blank period. Thirty trials were conducted for each stimulus in a pseudo-random order. By comparing the aperture conditions, the eccentricity and radial location of neurons’ receptive fields were estimated (Figures 1B,C). To obtain a retinotopy map for radial positions (Figure 1B), an expected value was calculated for each pixel using the following formula:

Σ r(i)×f(i)/Σ(f(i))

where r(i) is the radius of the annulus stimulus conditions (3.75, 8.75, or 14.00° in three conditions) and f(i) is the average ΔF/F across trials in the same condition. The map was smoothed with a circular averaging filter with a 5-pixel radius. Similarly, a retinotopy map for angular positions was calculated using the wedge stimulus conditions (Figure 1C). The stimulus presentation was controlled by Psychotoolbox-3^1^ on Matlab (R2014b, Mathworks, Inc., Natick, MA, USA) which also delivered trigger signals to an image acquisition PC.

Virus injections were made to MT regions representing near, but not in, the central visual field (green tracer, case 1; Figures 1B,C) and a peripheral and lower visual field (red tracer, case 2) in marmoset 1, and a peripheral visual field around the horizontal meridian in marmoset 2 (red tracer, case 3). Adeno-associated viral (AAV) tracers, which work as anterograde tracers by expressing fluorescent proteins in infected neurons, were injected into each designated site through a glass-pipette attached to an injector (Nanoject II, Drummond Scientific Company, Broomall, PA, USA). Two minutes after positioning of the pipette tip at a depth of 800 μm from the cortical surface, 200 nl of a viral tracer was injected at a rate of 25 nl/min. The tracers were a mixture of AAV1-Thy1S-tTA (1 × 10^9^ vector genomes (vg)/μl) and AAV1-TRE-clover (5 × 10^9^ vg/μl) for green fluorescent protein and AAV1-Thy1S-tTA (1 × 10^9^ vg/μl) and AAV1-TRE3-tdTomato (5 × 10^9^ vg/μl) for red fluorescent protein. After the tracer injection, the bone was replaced directly over the cortex after carefully ensuring that there was no ongoing bleeding. The bone was sealed with thin dental cement and the skin was sutured. After careful monitoring of recovery, the marmoset was returned to the cage.

### Histology and Brain Section Images

After a 3-week waiting period, the marmosets were perfused with 4% paraformaldehyde with picric acid (Abe et al., 2017) after the injection of atropine (0.15 μg/kg intramuscular), ketamine hydrochloride (25 mg/kg intramuscular), and an overdose of sodium pentobarbital (Somnopentyl, 100 mg/kg intraperitoneal; Kyoritsu Seiyaku, Tokyo, Japan). Histological processing was performed as previously described (Suzuki et al., 2015a; Abe et al., 2017; Miyakawa et al., 2017). Brain sections were sliced at a thickness of 50 μm and divided into 3 series for fluorescent image scanning, myelin staining (Pistorio et al., 2006), and Nissl substance staining with thionin (Suzuki et al., 2015a) in an interleaving manner. Brain section images were acquired using a slide scanner (NanoZoomer 2.0-HT, Hamamatsu Photonics K.K., Hamamatsu, Japan; 20× objective, 455 nm/pixel) with a filter cube (LED-DA/FI/TR/Cy5–4X-A-OMF, Semrock, Inc., Rochester, NY, USA), and injection sites were examined using an epi-fluorescence microscope (BZ-X700, Keyence, Osaka, Japan; Figure 1D). During sectioning, a pre-sectioning block-face brain image was taken of each section. Those images were used as references to reconstruct 3D brain section-derived images (Abe et al., 2017), which were rendered using Fluorender (Wan et al., 2012). Brain areas were identified based on the histologically stained section images according to a marmoset brain atlas (Paxinos et al., 2012).

### Flat Map Construction

A 3D mid-depth cortical surface representation of the gray matter was created. Pia and white matter contours were manually drawn on reconstructed histologically stained section images using graphics software (CorelDRAW X7, Ottawa, ON, Canada), and saved in scalable vector graphics format. Using those contours as boundary conditions, Laplace’s equations were solved to obtain “potential” (depth information) in the gray matter (e.g., Allen Mouse Common Coordinate Framework). Mid-potential (depth) points were collected to create a mid-depth cortical surface (MyCrustOpen Matlab function^2^). The obtained 3D surface was flattened by initial Tutte embedding (compute_parameterization function^3^) and the DMflatten algorithm, which reportedly yields more accurate flat maps than other flattening algorithms (Balasubramanian et al., 2010) by calculating exact geodesics on polyhedral surfaces (Balasubramanian et al., 2009). Each voxel of the 3D-reconstructed fluorescent signals was projected onto the mid-depth cortical surface based on the gradients of the potential. The projected fluorescent signals and identified brain areas were assigned to each polygon of the surface, and color coded. Custom Matlab scripts were used unless otherwise indicated.

## Results

Based on *in vivo* optical intrinsic signal imaging (Figures 1A–C), the virus tracers were injected into MT regions representing near the central field (case 1), a peripheral lower visual field (case 2) and a peripheral visual field around the horizontal meridian (case 3). The spreads of all injection sites were ~1 mm in diameter when examined in histological sections (Figure 1D).

### Projections to the Occipital Cortex

**Figure 2:**
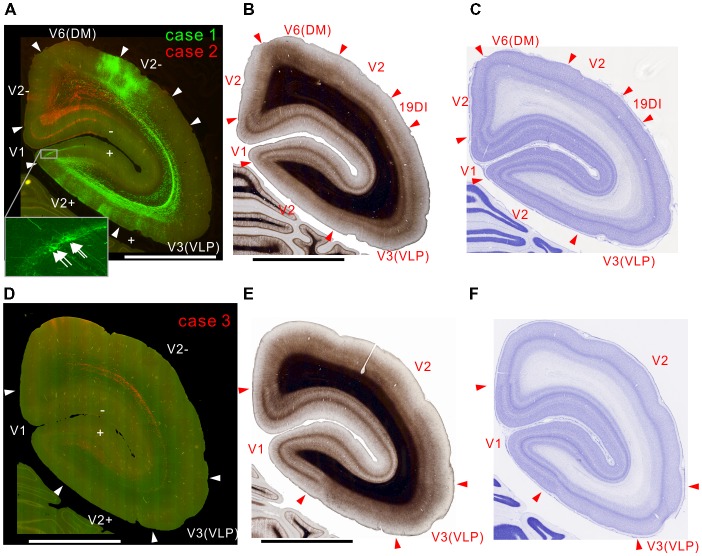
Brain section images at levels of the occipital cortex. **(A)** Fluorescent section image from cases 1 (green) and 2 (red). Green and red channel images taken with FITC and TRITC filters were overlaid. The + and − symbols indicate receptive field locations in the upper and lower visual fields, respectively, for each visual cortical area (Paxinos et al., 2012). Inset shows a higher magnification view. Some retrogradely labeled cells in V1 were indicated by arrows. **(B)** Myelin-stained section image. **(C)** Nissl substance-stained section image. **(D–F)** Images of brain sections from case 3 (red) slightly caudal from the sections in **(A–C)** shown in the same format as in **(A–C)** except that a far-red channel image (Cy5 filter) was used as a green channel and overlaid to enhance visibility of the projection by making the background autofluorescence appear yellowish, as in **(A)**. The triangles indicate brain area borders. The bars indicate 5 mm.

In case 2, there were projections to layer 6 of the corresponding lower visual field locations in V1 and V2, but not to the upper visual field (Figure 2A). In case 1, labeling was mainly in the upper visual field, but there was also some in lower visual fields (Figure 2A). This was presumably because the tracer injection was made in a site representing near the central visual field extending to both upper and lower visual fields. In V1, both injections elicited strong labeling in layers 1, 4B, and 6 (Figure 2A), consistent with previous studies (Maunsell and van Essen, 1983b; Ungerleider and Desimone, 1986b; Krubitzer and Kaas, 1990). The projection to layer 4B might also contain retrograde labeling to an extent, because there was a small number of infected cell bodies in layer 4B in V1 (Figure 2A inset), which is known to send strong projections to MT (Ungerleider and Desimone, 1986b; Rosa et al., 1993), and minor retrograde labeling occurs with both AAV and biotin dextran amine tracers (Wang et al., 2014). In V2, there were columnar projections with weaker labeling in layer 4 (Figure 2A), which may correspond to thick stripes in V2.

In case 3, consistent with the above-described cases, there were projections to layer 6 of V2 and V1 (Figure 2D). Because the site of this injection was an MT region representing an area around the horizontal meridian, the injection labeled axons going to both upper and lower visual fields (Figure 2D). The seemingly weaker projection may be because the volume of infected neurons was smaller than in the other cases (Figure 1D), although the same amount of tracer was injected.

**Figure 3:**
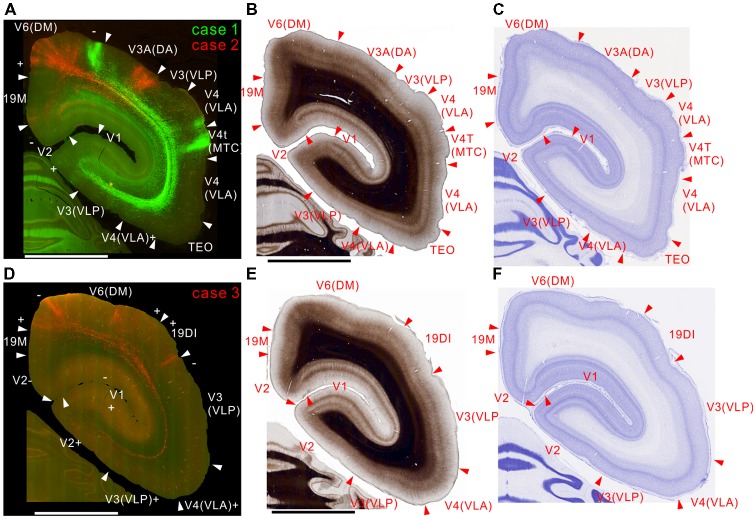
Brain section images at caudal levels from the injection sites, utilizing the same format as in Figure 2. **(A)** Fluorescent section image from case 1 (green) and 2 (red). **(B)** Image of the corresponding myelin-stained section. **(C)** Images of the corresponding Nissl substance-stained section. **(D–F)** Images of brain sections from case 3.

**Figure 4:**
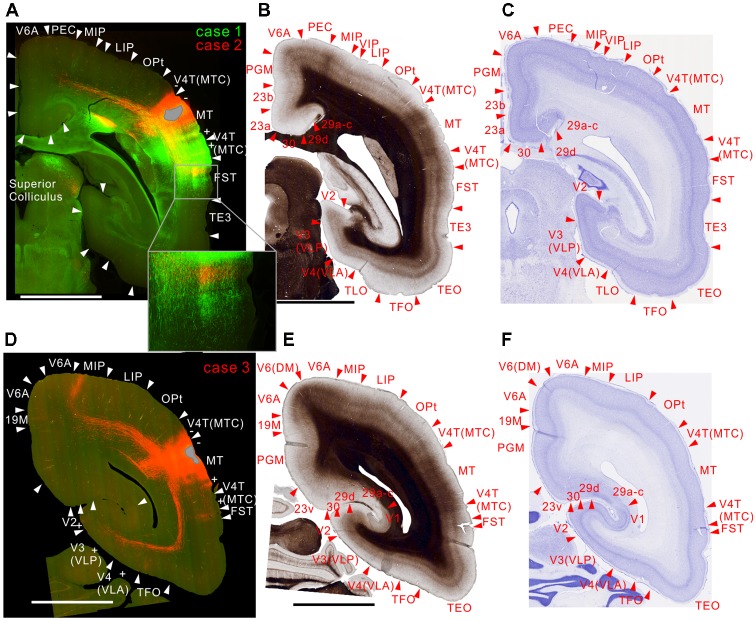
Brain section images at levels of the injection sites showing projections to intraparietal areas, utilizing the same format as in Figure 2. **(A)** Fluorescent section image from case 1 (green) and 2 (red). **(B)** Image of the corresponding myelin-stained section. **(C)** Images of the corresponding Nissl substance-stained section. **(D–F)** Images of brain sections from case 3. Inset in **(A)** is a higher magnification view showing horizontal connection in gray matter. In **(A)** the centers of the injection sites were slightly caudal from this section, whereas it was slightly rostral from this section in **(D)**.

All injection sites had projections to V3, V3A (DA) and V4 (VLA; Figures 2–4). Overall the projections to the occipital visual areas targeted supragranular layers and layer 6, with weaker labeling in layer 4 (except layer 4B in V1), suggesting feedback-type connections (Rockland and Pandya, 1979; Maunsell and van Essen, 1983b).

There were strong projections from MT to V6 (DM) in all the injections (Figures 2, 3). Those projections targeted all layers. In case 2, labeling was found in both upper and lower quadrant visual fields located in V6, consistent with the fact that V6 covers the entire contralateral visual fields (Galletti et al., 1999).

### Projections to the Temporal Cortex

**Figure 5:**
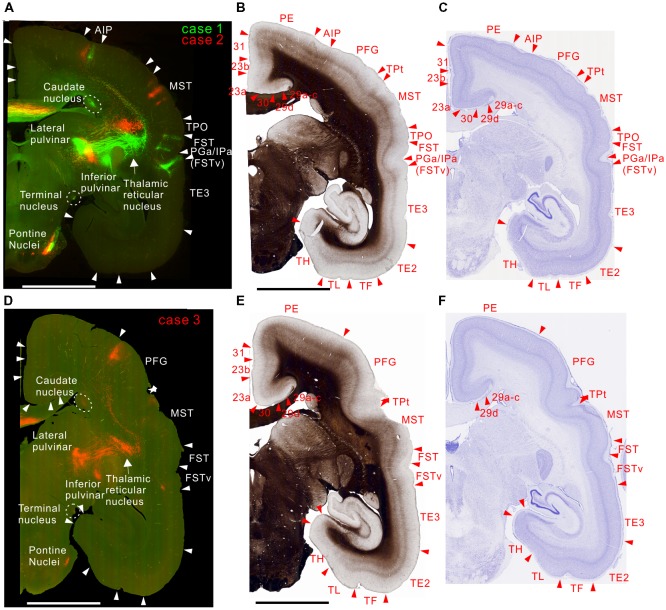
Brain section images at levels of rostral from the injection sites, utilizing the same format as in Figure 2. **(A)** Fluorescent section image from case 1 (green) and 2 (red). **(B)** Image of the corresponding myelin-stained section. **(C)** Images of the corresponding Nissl substance-stained section. **(D–F)** Images of brain sections from case 3.

MT projected to its surrounding areas such as MST, V4T (MTC), FST, FSTv (PGa/IPa), and TE 3 (Figures 3A, 4A,D, 5A,D). Those projections traveled in the white matter. Notably however, there were also horizontal connections to those areas near the injection sites that did not extend into the white matter (Figure 4A, inset). In a more rostral section, there were columnar patches in MST and FST (Figure 5A). These projections mainly targeted layer 4 and supragranular layers near the injection sites (Figures 3A, 4A,D, 5A,D). These projections are suggestive of feedforward-type connections (Rockland and Pandya, 1979; Maunsell and van Essen, 1983b).

### Projections to the Parietal Cortex

For intraparietal areas, there were projections to LIP, MIP and AIP (Figures 4, 5). These targeted all layers (Figures 4A,D), and there was a tendency toward stronger labeling in superficial layers (Figure 5A). In the marmoset that received two tracer injections, labeling was found in the same area but separated in AIP (Figure 5A), suggesting that there is a topographic relationship between MT projections and AIP.

### Projections to the Frontal Cortex

**Figure 6:**
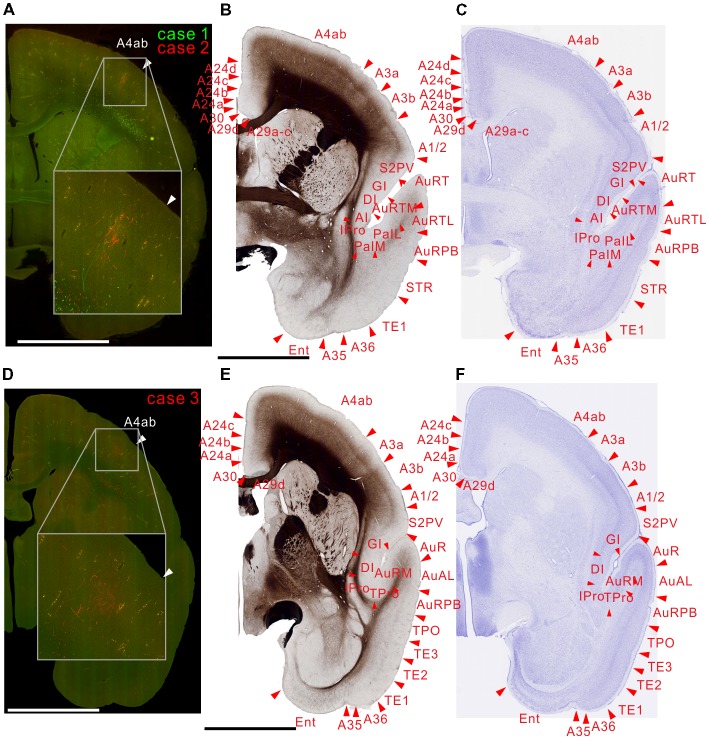
Brain section images at levels of the motor cortex, utilizing the same format as in Figure 2.

**Figure 7:**
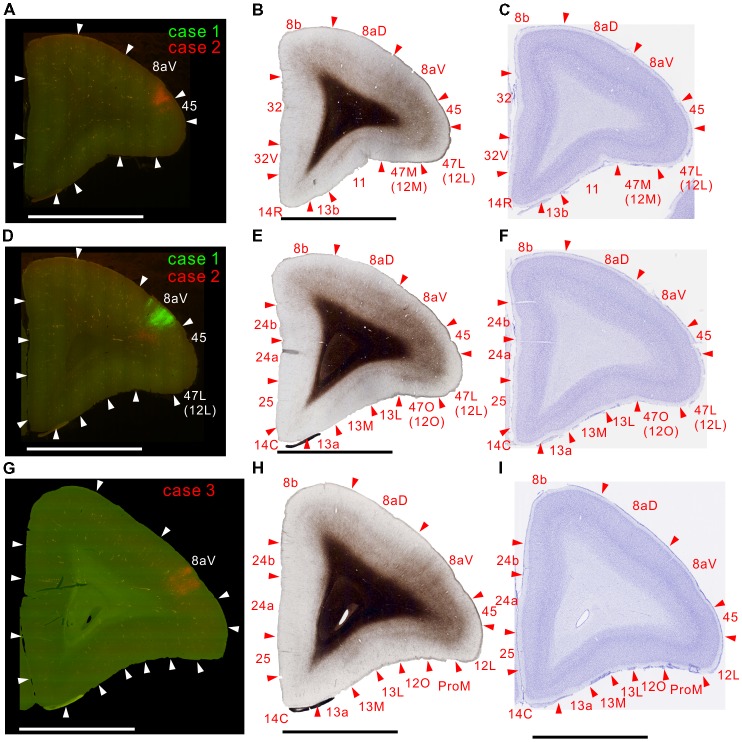
Brain section images at levels of the prefrontal cortex. **(A,D,G)** Fluorescent section images from cases 2 (red; **A**), 1 (green; **C**) and 3 (red; **E**). **(B,E,H)** Images of the next myelin-stained sections. **(C,F,I)** Images of the Nissl substance-stained section.

**Figure 8:**
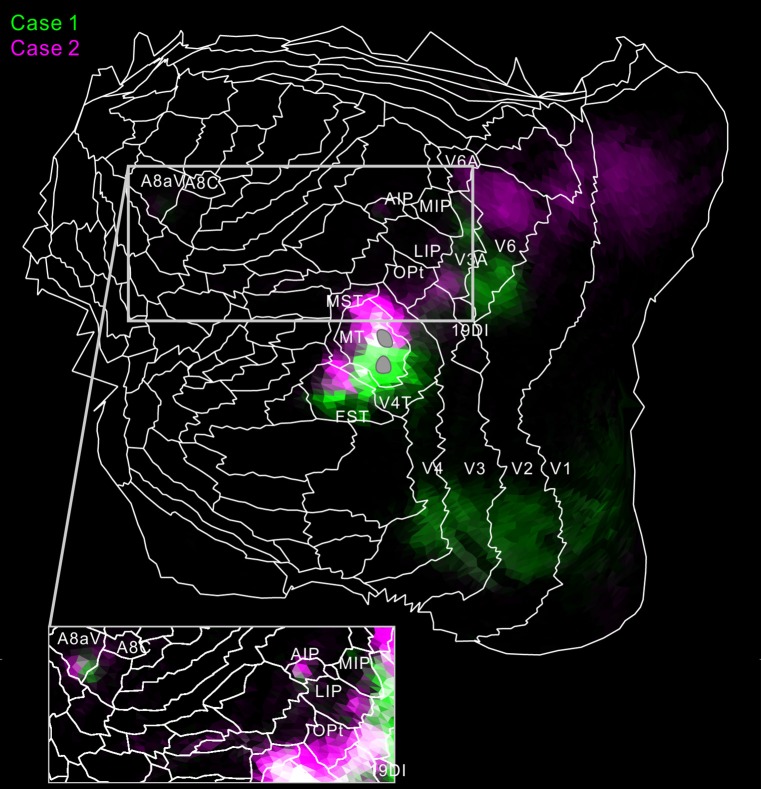
Flat map showing MT projection in case 1 (green) and 2 (magenta). To increase visibility, magenta, not red, was used for case 2. White regions indicate overlaps of the two tracer projections. The inset shows a part of the flat map in increased brightness. The gray regions indicate injection sites in MT.

**Figure 9:**
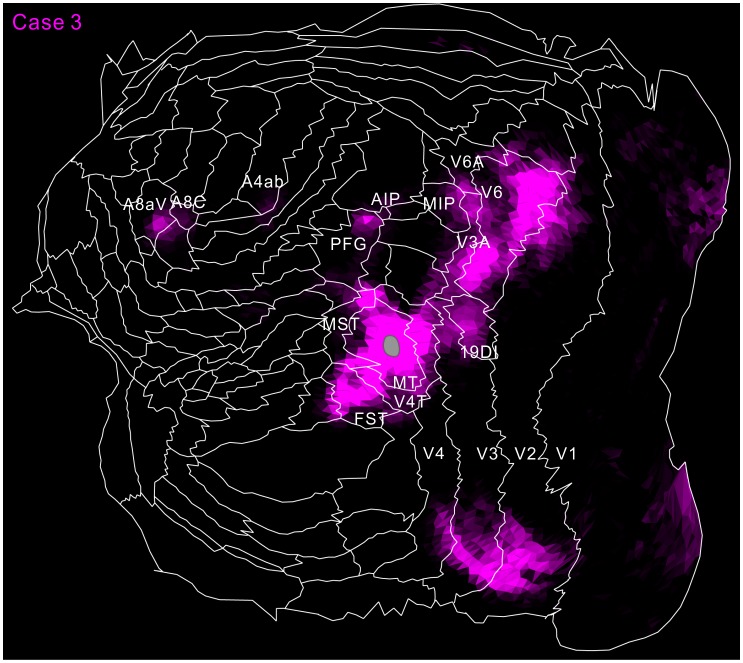
Flat map showing MT projection in case 3 (magenta). The same format as in Figure 8.

There were weak projections to A4ab, and prefrontal projections in A8C and A8aV in all injections (Figures 6–9). The projection to A8aV was consistent with a previous study (Reser et al., 2013), and mainly targeted layer 4 and supragranular layers. This area is known to have frontal eye fields and projections back to MT (Burman et al., 2006). Interestingly, as with AIP, labeling was separated between the tracers suggesting topographic projections from MT to A8aV (Figures 7, 8).

### Projections to Subcortical Brain Structures

In subcortical structures, MT projections were found in the superior colliculus (Figure 4A), caudate nucleus, lateral and inferior pulvinar nuclei and pontine nuclei (Figure 5) in all injections.

### Axon Fibers and Callosal Connection

**Figure 10:**
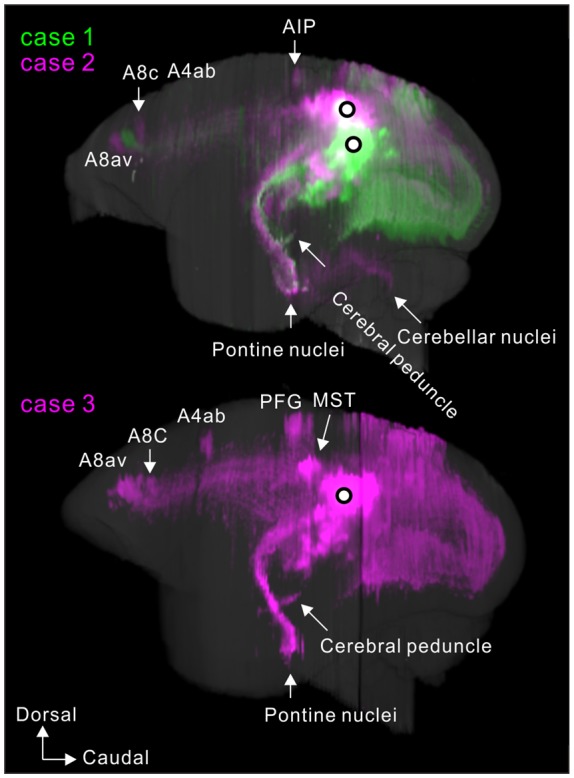
Lateral view of 3D reconstructions showing MT projection. The dots indicate injection sites.

**Figure 11:**
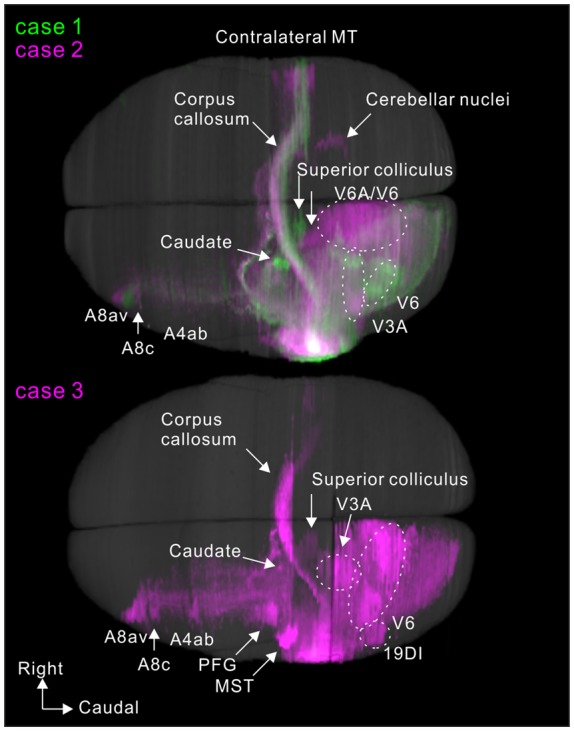
Dorsal view of 3D reconstructions showing MT projection.

**Figure 12:**
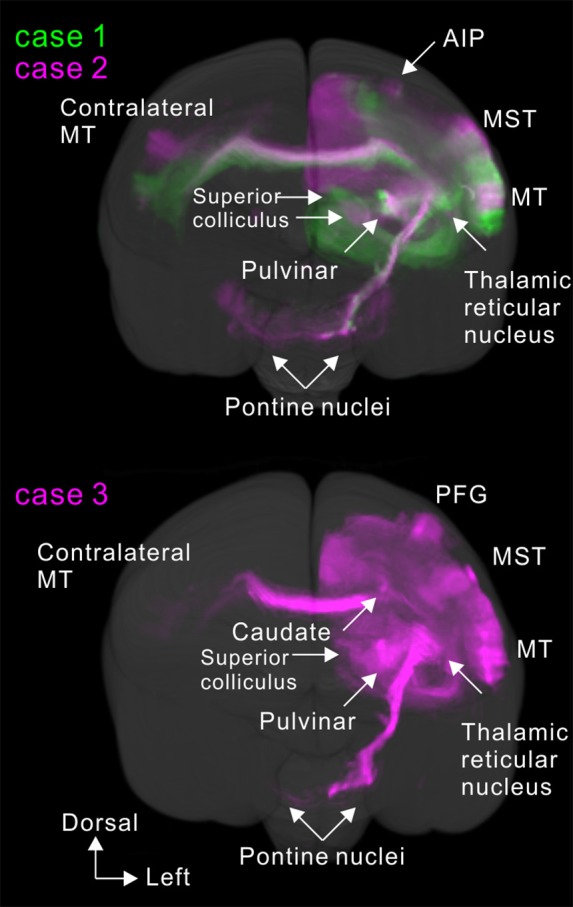
Rostral view of 3D reconstruction showing MT projection.

To visualize overall projection patterns, 3D reconstructions of the axonal projections were generated using the brain section images (Figures 10–12). MT had axon bundles in the white matter separately projecting to the prefrontal cortex, temporal cortex (with horizontal connection in the gray matter), and parietal and occipital cortexes, MT in the other hemisphere through the corpus callosum, superior colliculus, thalamic reticular nucleus, pulvinar, caudate nucleus and pontine nuclei.

## Discussion

**Figure 13:**
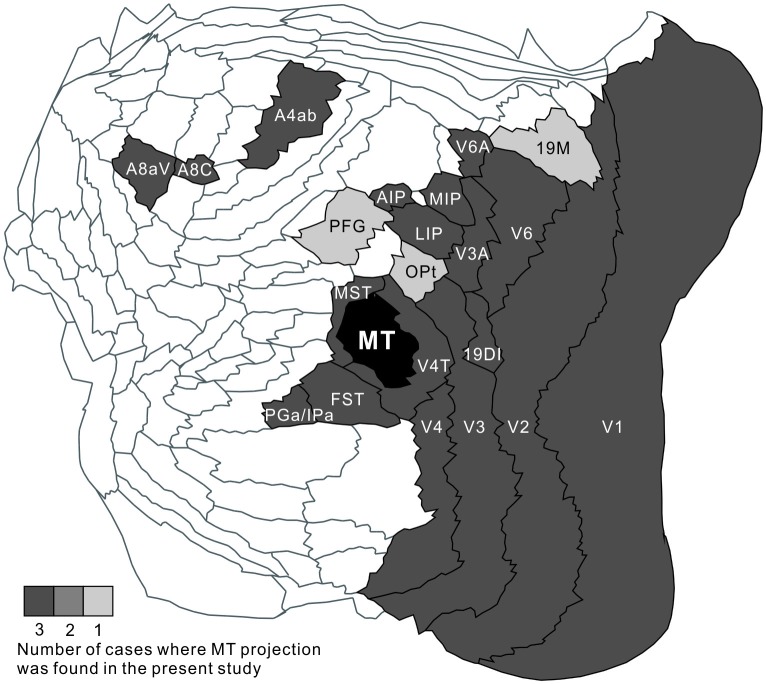
A summary flat map showing consistency between injections. For each brain area, the number of cases where MT projection was found was indicated by gray scale.

MT projected to nearby temporal areas, MST, FST, FSTv (PGa/IPa), the occipital visual areas V1, V2, V3 (VLP), V4 (VLA), V4T (MTC), the dorsal visual pathway V3A (DA), parietal V6, V6A, intraparietal AIP, MIP, LIP and frontal A4ab, prefrontal A8aV and A8C in all tracer injections (Figure 13). New findings in this study are that there was MT projection to V6 (DM), A4ab and topographic MT projections to AIP and A8aV in marmosets.

### Comparison With Previous Tracing Studies in Marmosets

Most of the projection target areas identified were consistent with a previous study using peroxidase-conjugated wheat germ agglutinin (WGA-HRP), in which anterograde connections between MT and MTC, FST, MST and occipital visual areas were detected in marmosets (Krubitzer and Kaas, 1990). However, there were differences. In that previous study, projections to V6 (DM) and A8aV (FEF) were not detected. This may be due to a technical difference such as the real flattening procedure and/or the WGA-HRP method used in the previous study. Notably, thin axons are difficult to observe using WGA-HRP. Without additional information or access to the raw data obtained in the previous study, it is hard to reconcile with the results of the current study. In the current study there were clear projections to V6 (DM), A4ab and A8aV with traveling axons in the white matter in all three injections (Figures 3, 6, 7, 10–12). In addition, in a previous retrograde tracer study MT reportedly received input from both V6 (DM) and A8aV (Rosa et al., 2009). Another previous study compared anatomical input from MT in macaques, marmosets and capuchin monkeys, and found that V4T (MTC), MST, FST, V6 (DM), dorsal posterior parietal cortex (LIP, VIP), V3 (VLP), V4 (VLA) and A8aV received anatomical input from MT similarly across all three species (Palmer and Rosa, 2006). Those are the areas that MT projected to in the present study. This demonstrates that these connections were well conserved during evolution.

### Comparison With Macaque Studies

Many studies have investigated MT connections in macaques. Those studies have found reciprocal connections with V1 (Maunsell and van Essen, 1983b; Weller and Kaas, 1983; Perkel et al., 1986; Van Essen et al., 1986; Ungerleider and Desimone, 1986a; Rockland, 1989; Shipp and Zeki, 1989; Sincich and Horton, 2003), V2 (Maunsell and van Essen, 1983b; DeYoe and Van Essen, 1985; Ungerleider and Desimone, 1986a; Rockland, 1995; Stepniewska and Kaas, 1996; Anderson and Martin, 2002), V3 and V3A (Maunsell and van Essen, 1983b; Ungerleider and Desimone, 1986a; Felleman et al., 1997), V4 and V4T (MTC; Maunsell and van Essen, 1983b; Desimone and Ungerleider, 1986; Ungerleider and Desimone, 1986a), V6 (Galletti et al., 2001; Passarelli et al., 2011), MST (Maunsell and van Essen, 1983b; Desimone and Ungerleider, 1986; Ungerleider and Desimone, 1986a), FST (Desimone and Ungerleider, 1986; Ungerleider and Desimone, 1986a; Boussaoud et al., 1990), VIP (Maunsell and van Essen, 1983b; Ungerleider and Desimone, 1986a; Boussaoud et al., 1990), LIP (Blatt et al., 1990) and FEF (Ungerleider and Desimone, 1986a; Leichnetz, 1989; Stanton et al., 1995; Markov et al., 2014). Thus, those findings were consistent with the MT projections detected in marmosets, using anterograde tracers, in the present study.

In macaques, MT projections to TEa and TEm (Seltzer and Pandya, 1989), TE3 and TEO (Seltzer and Pandya, 1991), POa (Ungerleider and Desimone, 1986a; Seltzer and Pandya, 1991), and PG and 7a (Neal et al., 1990) have been reported. Although no such projections were detected in the marmosets in the present study, macaque area POa (Seltzer and Pandya, 1991) corresponds to LIP (which was labeled in the marmoset), and PO (Ungerldeider and Desimone, 1986) is actually part of area V6 (DM; see Angelucci and Rosa, 2015). Therefore, detailed comparisons are hampered by the use of different nomenclature in addition to unclear homologies between the species. Previous studies with both anterograde and retrograde tracers have detected connections with TE in marmosets (Krubitzer and Kaas, 1990; Palmer and Rosa, 2006).

With regard to subcortical connections, reciprocal connections with the pulvinar (Standage and Benevento, 1983; Ungerleider et al., 1984) as well as projections to the superior colliculus (Maioli et al., 1992), lateral basal nucleus amygdala (Iwai and Yukie, 1987), thalamic reticular nucleus, caudate, putamen, claustrum (Ungerleider et al., 1984), nucleus of the optic tract, dorsal terminal nucleus and dorsolateral pontine nucleus (Distler and Hoffmann, 2001; Distler et al., 2002) have been reported. In the present study, we also found MT projections to the pulvinar, superior colliculus, thalamic reticular nucleus, caudate, terminal nucleus and pontine nucleus in marmosets.

## Conclusion

Using a combination of new virus tracers and *in vivo* optical signal imaging, we found that MT projects to occipital visual areas and its surrounding areas in the temporal cortex, as well as to the dorsal visual pathway, intraparietal areas and prefrontal cortex. The different injections into MT exhibited similarity in the distribution of labeling throughout the brain, which resembled that observed in retrograde tracer studies that mapped projections to MT (Palmer and Rosa, 2006), suggesting that these connections are reciprocal.

## Author Contributions

HA, TT and NI contributed to the experimental design, data interpretation and writing of the manuscript. HA, TT, TH, SW and WS performed the marmoset experiments. HMashiko, NK and KS performed the histological experiments. HMizukami, AW and TY contributed to the AAV tracers.

## Conflict of Interest Statement

The authors declare that the research was conducted in the absence of any commercial or financial relationships that could be construed as a potential conflict of interest.

## Footnotes

^1^http://psychtoolbox.org/

^2^https://jp.mathworks.com/matlabcentral/fileexchange/63731

^3^https://jp.mathworks.com/matlabcentral/fileexchange/5355

## References

[B1] Abe H. Tani T. Mashiko H. Kitamura N. Miyakawa N. Mimura K.. (2017). 3D reconstruction of brain section images for creating axonal projection maps in marmosets. J. Neurosci. Methods 286, 102–113. 10.1016/j.jneumeth.2017.04.016 28577985

[B2] Albright T. D. (1984). Direction and orientation selectivity of neurons in visual area MT of the macaque. J. Neurophysiol. 52, 1106–1130. 10.1152/jn.1984.52.6.1106 6520628

[B3] Allman J. M. Kaas J. H. (1971). A representation of the visual field in the caudal third of the middle tempral gyrus of the owl monkey (*Aotus trivirgatus*). Brain Res. 31, 85–105. 10.1016/0006-8993(71)90635-4 4998922

[B4] Anderson J. C. Martin K. A. (2002). Connection from cortical area V2 to MT in macaque monkey. J. Comp. Neurol. 443, 56–70. 10.1002/cne.10100 11793347

[B5] Angelucci A. Rosa M. G. (2015). Resolving the organization of the third tier visual cortex in primates: a hypothesis-based approach. Vis. Neurosci. 32:E010. 10.1017/s0952523815000073 26241792 PMC5301954

[B6] Balasubramanian M. Polimeni J. R. Schwartz E. L. (2009). Exact geodesics and shortest paths on polyhedral surfaces. IEEE Trans. Pattern Anal. Mach. Intell. 31, 1006–1016. 10.1109/tpami.2008.213 19372606

[B7] Balasubramanian M. Polimeni J. R. Schwartz E. L. (2010). Near-isometric flattening of brain surfaces. Neuroimage 51, 694–703. 10.1016/j.neuroimage.2010.02.008 20149886 PMC2856738

[B8] Blatt G. J. Andersen R. A. Stoner G. R. (1990). Visual receptive field organization and cortico-cortical connections of the lateral intraparietal area (area LIP) in the macaque. J. Comp. Neurol. 299, 421–445. 10.1002/cne.902990404 2243159

[B9] Born R. T. Bradley D. C. (2005). Structure and function of visual area MT. Annu. Rev. Neurosci. 28, 157–189. 10.1146/annurev.neuro.26.041002.131052 16022593

[B11] Boussaoud D. Ungerleider L. G. Desimone R. (1990). Pathways for motion analysis: cortical connections of the medial superior temporal and fundus of the superior temporal visual areas in the macaque. J. Comp. Neurol. 296, 462–495. 10.1002/cne.902960311 2358548

[B12] Britten K. H. Newsome W. T. Shadlen M. N. Celebrini S. Movshon J. A. (1996). A relationship between behavioral choice and the visual responses of neurons in macaque MT. Vis. Neurosci. 13, 87–100. 10.1017/s095252380000715x 8730992

[B14] Burman K. J. Palmer S. M. Gamberini M. Rosa M. G. (2006). Cytoarchitectonic subdivisions of the dorsolateral frontal cortex of the marmoset monkey (*Callithrix jacchus*) and their projections to dorsal visual areas. J. Comp. Neurol. 495, 149–172. 10.1002/cne.20837 16435289

[B15] Burman K. J. Palmer S. M. Gamberini M. Spitzer M. W. Rosa M. G. (2008). Anatomical and physiological definition of the motor cortex of the marmoset monkey. J. Comp. Neurol. 506, 860–876. 10.1002/cne.21580 18076083

[B16] Cardin V. Smith A. T. (2010). Sensitivity of human visual and vestibular cortical regions to egomotion-compatible visual stimulation. Cereb. Cortex 20, 1964–1973. 10.1093/cercor/bhp268 20034998 PMC2901022

[B100] Chen S. C. Morley J. W. Solomon S. G. (2015). Spatial precision of population activity in primate area MT. J. Neurophysiol. 114, 869–878. 10.1152/jn.00152.2015 26041825 PMC4533107

[B17] Desimone R. Ungerleider L. G. (1986). Multiple visual areas in the caudal superior temporal sulcus of the macaque. J. Comp. Neurol. 248, 164–189. 10.1002/cne.902480203 3722457 PMC11528348

[B18] DeYoe E. A. Van Essen D. C. (1985). Segregation of efferent connections and receptive field properties in visual area V2 of the macaque. Nature 317, 58–61. 10.1038/317058a0 2412132

[B19] Distler C. Hoffmann K. P. (2001). Cortical input to the nucleus of the optic tract and dorsal terminal nucleus (NOT-DTN) in macaques: a retrograde tracing study. Cereb. Cortex 11, 572–580. 10.1093/cercor/11.6.572 11375918

[B20] Distler C. Mustari M. J. Hoffmann K. P. (2002). Cortical projections to the nucleus of the optic tract and dorsal terminal nucleus and to the dorsolateral pontine nucleus in macaques: a dual retrograde tracing study. J. Comp. Neurol. 444, 144–158. 10.1002/cne.10127 11835187

[B21] Dubner R. Zeki S. M. (1971). Response properties and receptive fields of cells in an anatomically defined region of the superior temporal sulcus in the monkey. Brain Res. 35, 528–532. 10.1016/0006-8993(71)90494-x 5002708

[B23] Felleman D. J. Burkhalter A. Van Essen D. C. (1997). Cortical connections of areas V3and VP of macaque monkey extrastriate visual cortex. J. Comp. Neurol. 379, 21–47. 10.1002/(sici)1096-9861(19970303)379:1<21::aid-cne3>3.0.co;2-k 9057111

[B22] Felleman D. J. Kaas J. H. (1984). Receptive-field properties of neurons in middle temporal visual area (MT) of owl monkeys. J. Neurophysiol. 52, 488–513. 10.1152/jn.1984.52.3.488 6481441

[B24] Galletti C. Fattori P. Gamberini M. Kutz D. F. (1999). The cortical visual area V6: brain location and visual topography. Eur. J. Neurosci. 11, 3922–3936. 10.1046/j.1460-9568.1999.00817.x 10583481

[B25] Galletti C. Gamberini M. Kutz D. F. Fattori P. Luppino G. Matelli M. (2001). The cortical connections of area V6: an occipito-parietal network processing visual information. Eur. J. Neurosci. 13, 1572–1588. 10.1046/j.0953-816x.2001.01538.x 11328351

[B27] Iwai E. Yukie M. (1987). Amygdalofugal and amygdalopetal connections with modality-specific visual cortical areas in macaques (*Macaca fuscata, M. mulatta* and *M. fascicularis*). J. Comp. Neurol. 261, 362–387. 10.1002/cne.902610304 3611417

[B28] Krubitzer L. A. Kaas J. H. (1990). Cortical connections of MT in four species of primates: areal, modular, and retinotopic patterns. Vis. Neurosci. 5, 165–204. 10.1017/s0952523800000213 2278944

[B29] Leichnetz G. R. (1989). Inferior frontal eye field projections to the pursuit-related dorsolateral pontine nucleus and middle temporal area (MT) in the monkey. Vis. Neurosci. 3, 171–180. 10.1017/s0952523800004478 2487099

[B31] Lui L. L. Rosa M. G. (2015). Structure and function of the middle temporal visual area (MT) in the marmoset: comparisons with the macaque monkey. Neurosci. Res. 93, 62–71. 10.1016/j.neures.2014.09.012 25304293

[B32] Maioli M. G. Domeniconi R. Squatrito S. Riva Sanseverino E. (1992). Projections from cortical visual areas of the superior temporal sulcus to the superior colliculus, in macaque monkeys. Arch. Ital. Biol. 130, 157–166. 1510547

[B33] Markov N. T. Ercsey-Ravasz M. M. Ribeiro Gomes A. R. Lamy C. Magrou L. Vezoli J.. (2014). A weighted and directed interareal connectivity matrix for macaque cerebral cortex. Cereb. Cortex 24, 17–36. 10.1093/cercor/bhs270 23010748 PMC3862262

[B34] Maunsell J. H. van Essen D. C. (1983a). Functional properties of neurons in middle temporal visual area of the macaque monkey. I. Selectivity for stimulus direction, speed, and orientation. J. Neurophysiol. 49, 1127–1147. 10.1152/jn.1983.49.5.1127 6864242

[B35] Maunsell J. H. van Essen D. C. (1983b). The connections of the middle temporal visual area (MT) and their relationship to a cortical hierarchy in the macaque. J. Neurosci. 3, 2563–2586. 10.1523/JNEUROSCI.03-12-02563.1983 6655500 PMC6564662

[B37] Miyakawa N. Banno T. Abe H. Tani T. Suzuki W. Ichinohe N. (2017). Representation of glossy material surface in ventral superior temporal sulcal area of common marmosets. Front. Neural Circuits 11:17. 10.3389/fncir.2017.00017 28367117 PMC5355424

[B38] Neal J. W. Pearson R. C. Powell T. P. (1990). The connections of area PG, 7a, with cortex in the parietal, occipital and temporal lobes of the monkey. Brain Res. 532, 249–264. 10.1016/0006-8993(90)91767-b 2282518

[B39] Newsome W. T. Paré E. B. (1988). A selective impairment of motion perception following lesions of the middle temporal visual area (MT). J. Neurosci. 8, 2201–2211. 10.1523/JNEUROSCI.08-06-02201.1988 3385495 PMC6569328

[B40] Palmer S. M. Rosa M. G. (2006). Quantitative analysis of the corticocortical projections to the middle temporal area in the marmoset monkey: evolutionary and functional implications. Cereb. Cortex 16, 1361–1375. 10.1093/cercor/bhj078 16292001

[B41] Passarelli L. Rosa M. G. Gamberini M. Bakola S. Burman K. J. Fattori P.. (2011). Cortical connections of area V6Av in the macaque: a visual-input node to the eye/hand coordination system. J. Neurosci. 31, 1790–1801. 10.1523/JNEUROSCI.4784-10.2011 21289189 PMC6623732

[B42] Paxinos G. Watson C. Petrides M. Rosa M. Tokuno H. (2012). The Marmoset Brain in Stereotaxic Coordinates. London: Academic Press Inc.

[B43] Perkel D. J. Bullier J. Kennedy H. (1986). Topography of the afferent connectivity of area 17 in the macaque monkey: a double-labelling study. J. Comp. Neurol. 253, 374–402. 10.1002/cne.902530307 3793996

[B44] Pistorio A. L. Hendry S. H. Wang X. (2006). A modified technique for high-resolution staining of myelin. J. Neurosci. Methods 153, 135–146. 10.1016/j.jneumeth.2005.10.014 16310256

[B45] Pitzalis S. Bozzacchi C. Bultrini A. Fattori P. Galletti C. Di Russo F. (2013). Parallel motion signals to the medial and lateral motion areas V6 and MT^+^. Neuroimage 67, 89–100. 10.1016/j.neuroimage.2012.11.022 23186916

[B46] Reser D. H. Burman K. J. Yu H. H. Chaplin T. A. Richardson K. E. Worthy K. H.. (2013). Contrasting patterns of cortical input to architectural subdivisions of the area 8 complex: a retrograde tracing study in marmoset monkeys. Cereb. Cortex 23, 1901–1922. 10.1093/cercor/bhs177 22735155 PMC3698368

[B47] Rockland K. S. (1989). Bistratified distribution of terminal arbors of individual axons projecting from area V1 to middle temporal area (MT) in the macaque monkey. Vis. Neurosci. 3, 155–170. 10.1017/s0952523800004466 2487098

[B48] Rockland K. S. (1995). Morphology of individual axons projecting from area V2 to MT in the macaque. J. Comp. Neurol. 355, 15–26. 10.1002/cne.903550105 7636009

[B49] Rockland K. S. Pandya D. N. (1979). Laminar origins and terminations of cortical connections of the occipital lobe in the rhesus monkey. Brain Res. 179, 3–20. 10.1016/0006-8993(79)90485-2 116716

[B50] Rosa M. G. Palmer S. M. Gamberini M. Burman K. J. Yu H. H. Reser D. H.. (2009). Connections of the dorsomedial visual area: pathways for early integration of dorsal and ventral streams in extrastriate cortex. J. Neurosci. 29, 4548–4563. 10.1523/JNEUROSCI.0529-09.2009 19357280 PMC6665729

[B51] Rosa M. G. P. Soares J. G. Fiorani M. Jr. Gattass R. (1993). Cortical afferents of visual area MT in the Cebus monkey: possible homologies between New and Old Worldmonkeys. Vis. Neurosci. 10, 827–855. 10.1017/s0952523800006064 8217935

[B52] Salzman C. D. Britten K. H. Newsome W. T. (1990). Cortical microstimulation influences perceptual judgements of motion direction. Nature 346, 174–177. 10.1038/346174a0 2366872

[B53] Seltzer B. Pandya D. N. (1989). Intrinsic connections and architectonics of the superior temporal sulcus in the rhesus monkey. J. Comp. Neurol. 290, 451–471. 10.1002/cne.902900402 2482305

[B54] Seltzer B. Pandya D. N. (1991). Post-rolandic cortical projections of the superior temporal sulcus in the rhesus monkey. J. Comp. Neurol. 312, 625–640. 10.1002/cne.903120412 1761745

[B55] Shadlen M. N. Britten K. H. Newsome W. T. Movshon J. A. (1996). A computational analysis of the relationship between neuronal and behavioral responses to visual motion. J. Neurosci. 16, 1486–1510. 10.1523/JNEUROSCI.16-04-01486.1996 8778300 PMC6578557

[B56] Shipp S. Zeki S. (1989). The organization of connections between areas V5 and V1 in macaque monkey visual cortex. Eur. J. Neurosci. 1, 309–332. 10.1111/j.1460-9568.1989.tb00798.x 12106142

[B57] Sincich L. C. Horton J. C. (2003). Independent projection streams from macaque striate cortex to the second visual area and middle temporal area. J. Neurosci. 23, 5684–5692. 10.1523/JNEUROSCI.4833-13.2013 12843271 PMC6741268

[B58] Standage G. P. Benevento L. A. (1983). The organization of connections between the pulvinar and visual area MT in the macaque monkey. Brain Res. 262, 288–294. 10.1016/0006-8993(83)91020-x 6839157

[B59] Stanton G. B. Bruce C. J. Goldberg M. E. (1995). Topography of projections to posterior cortical areas from the macaque frontal eye fields. J. Comp. Neurol. 353, 291–305. 10.1002/cne.903530210 7745137

[B60] Stepniewska I. Kaas J. H. (1996). Topographic patterns of V2 cortical connections in macaque monkeys. J. Comp. Neurol. 371, 129–152. 10.1002/(sici)1096-9861(19960715)371:1<129::aid-cne8>3.0.co;2-5 8835723

[B61] Suzuki W. Banno T. Miyakawa N. Abe H. Goda N. Ichinohe N. (2015a). Mirror neurons in a new world monkey, common marmoset. Front. Neurosci. 9:459. 10.3389/fnins.2015.00459 26696817 PMC4674550

[B62] Suzuki W. Tani T. Banno T. Miyakawa N. Abe H. Ichinohe I. (2015b). Functional columns in superior temporal sulcus areas of the common marmoset. Neuroreport 26, 1133–1139. 10.1097/WNR.0000000000000483 26512934

[B101] Townsend R. G. Solomon S. S. Chen S. C. Pietersen A. N. Martin P. R. Solomon S. G.. (2015). Emergence of complex wave patterns in primate cerebral cortex. J. Neurosci. 35, 4657–4662. 10.1523/JNEUROSCI.4509-14.2015 25788682 PMC4363391

[B102] Townsend R. G. Solomon S. S. Martin P. R. Solomon S. G. Gong P. (2017). Visual Motion Discrimination by Propagating Patterns in Primate Cerebral Cortex. J. Neurosci. 37, 10074–10084. 10.1523/JNEUROSCI.1538-17.2017 28912155 PMC6596543

[B63] Ungerleider L. G. Desimone R. (1986a). Cortical connections of visual area MT in the macaque. J. Comp. Neurol. 248, 190–222. 10.1002/cne.902480204 3722458

[B64] Ungerleider L. G. Desimone R. (1986b). Projections to the superior temporal sulcus from the central and peripheral field representations of V1 and V2. J. Comp. Neurol. 248, 147–173. 10.1002/cne.902480202 3722456

[B65] Ungerleider L. G. Desimone R. Galkin T. W. Mishkin M. (1984). Subcortical projections of area MT in the macaque. J. Comp. Neurol. 223, 368–386. 10.1002/cne.902230304 6323553

[B67] Van Essen D. C. Newsome W. T. Maunsell J. H. Bixby J. L. (1986). The projections from striate cortex (V1) to areas V2 and V3 in the macaque monkey: asymmetries, areal boundaries, and patchy connections. J. Comp. Neurol. 244, 451–480. 10.1002/cne.902440405 3958238

[B68] Wan Y. Otsuna H. Chien C. B. Hansen C. (2012). “FluoRender: an application of 2D image space methods for 3D and 4D confocal microscopy data visualization in neurobiology research,” in Proceedings of the IEEE Pacific Visualization Symposium (Songdo: IEEE), 201–208. 10.1109/pacificvis.2012.6183592 PMC3622106 23584131

[B69] Wang Q. Henry A. M. Harris J. A. Oh S. W. Joines K. M. Nyhus J.. (2014). Systematic comparison of adeno-associated virus and biotinylated dextran amine reveals equivalent sensitivity between tracers and novel projection targets in the mouse brain. J. Comp. Neurol. 522, 1989–2012. 10.1002/cne.23567 24639291

[B70] Weller R. E. Kaas J. H. (1983). Retinotopic patterns of connections of area 17 with visual areas V-II and MT in macaque monkeys. J. Comp. Neurol. 220, 253–279. 10.1002/cne.902200302 6315784

[B103] Zavitz E. Yu H. H. Rowe E. G. Rosa M. G. Price N. S. C. (2016). Rapid adaptation induces persistent biases in population codes for visual motion. J. Neurosci. 36, 4579–4590. 10.1523/JNEUROSCI.4563-15.2016 27098699 PMC6601831

[B104] Zavitz E. Yu H. H. Rosa M. G. P. Price N. S. C. (2017). Correlated variability in the neurons with the strongest tuning improves direction coding. Cereb. Cortex 10.1093/cercor/bhx344 29300838

[B71] Zohary E. Shadlen M. N. Newsome W. T. (1994). Correlated neuronal discharge rate and its implications for psychophysical performance. Nature 370, 140–143. 10.1038/370140a0 8022482

